# Supplementary material for: Characterization of Severity in Zellweger Spectrum Disorder by Clinical Findings: A Scoping Review, Meta-Analysis and Medical Chart Review
Source: Cells. 2022 Jun 10;11(12):1891. doi: 10.3390/cells11121891 (PMC9221082; doi:10.3390/cells11121891)

**Supplementary Figure S1. Flowchart for phenotypic severity designation in individual patients with ZSD reported in the literature and from our natural history study**. The choice of features listed above for each phenotypic severity was based on published cardinal clinical features and on information collected from our natural history study (Braverman et al. 2016 PMID 26750748, Klouwer et al. 2015 PMID 26627182, *clinicaltrials.gov* identifier NCT01668186).

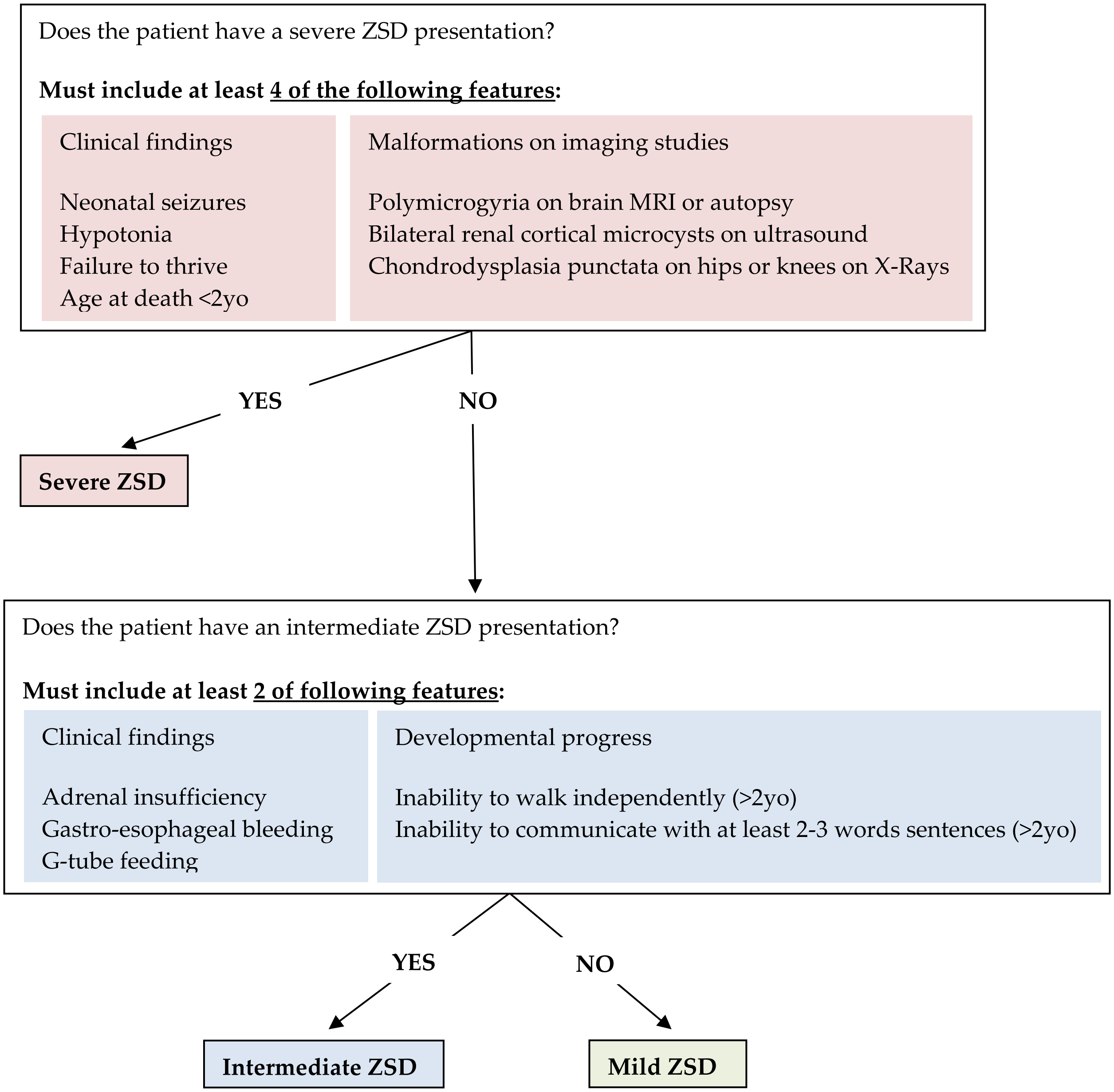

Supplement: Supplementary file 1 [file cells-11-01891-s001.zip › cells-1744944-supplementary/Supp_FigureS1_Severity_Flowchart.pdf]
