# Supplementary material for: Characterization of Severity in Zellweger Spectrum Disorder by Clinical Findings: A Scoping Review, Meta-Analysis and Medical Chart Review
Source: Cells. 2022 Jun 10;11(12):1891. doi: 10.3390/cells11121891 (PMC9221082; doi:10.3390/cells11121891)
Supplement: Supplementary file 1 [file cells-11-01891-s001.zip › cells-1744944-supplementary/Supp_FigureS2_Model_Symptom_below2yo.pdf]

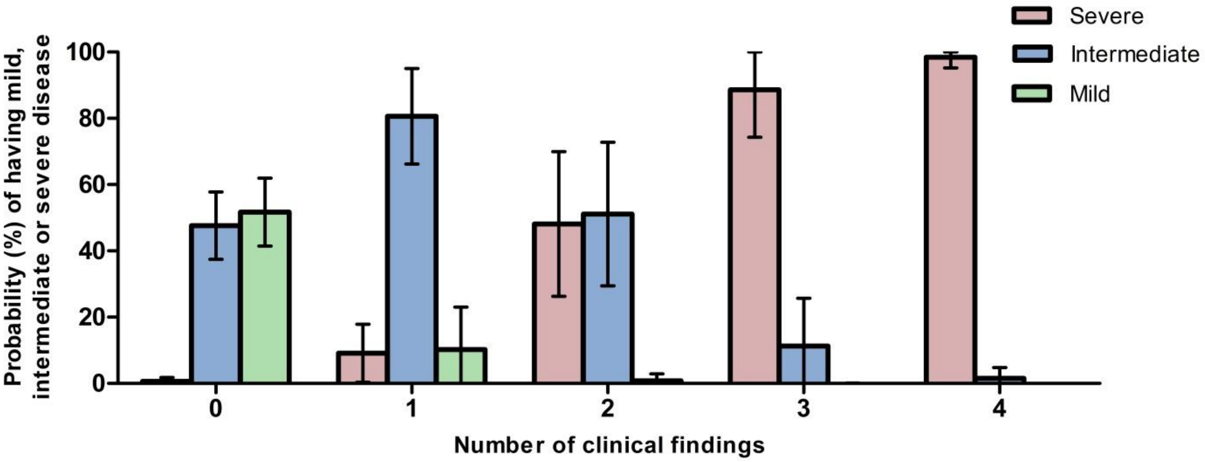

Supplementary Figure S2. Predicted probabilities of each severity category by number of clinical findings among seizure disorder, abnormal EEG, bilateral cortical microcysts and cardiac abnormalities, for findings present before the age of two years. The probabilities were calculated from a multinomial logistic model with 95% confidence intervals shown as the error bars.
