# Supplementary material for: Characterization of Severity in Zellweger Spectrum Disorder by Clinical Findings: A Scoping Review, Meta-Analysis and Medical Chart Review
Source: Cells. 2022 Jun 10;11(12):1891. doi: 10.3390/cells11121891 (PMC9221082; doi:10.3390/cells11121891)
Supplement: Supplementary file 1 [file cells-11-01891-s001.zip › cells-1744944-supplementary/Supp_TableS1_NHS_cohort_Demographics.pdf]

		n patients	% of cohort
**Gender**			
	Males	79	52.7
	Females	71	47.3
**Country / Continent**			
	United States	95	63.3
	Canada	28	18.7
	Western Europe	12	8
	Middle East	5	3.3
	Australia	5	3.3
	South America	2	1.3
	Eastern Europe	2	1.3
	India	1	0.7
**Genotype**			
	PEX1 null / null[1]	6	4
	PEX10 null / null	1	0.7
	PEX1 p.G843D / null	39	26
	PEX1 missense / null	5	3.3
	PEX6 missense / null	7	4.7
	Other PEX missense / null (PEX5, PEX10, PEX13, PEX16, PEX26)	10	6.7
	PEX1 p.G843D / p.G843D	23	15.3
	PEX1 missense / missense	8	5.3
	PEX6 missense / missense	6	4
	Other PEX missense / missense (PEX10, PEX12, PEX13, PEX16, PEX19, PEX26)	10	6.7
	PEX mutations not classified[2]	21	14
	Unknown	14	9.3

Supplementary Table S1. Demographics of ZSD patients in the natural history cohort.
[1]Null alleles are defined as non-functional alleles including nonsense mutations, frameshift and subsequent premature stop codon, or splice site mutations. [2]Mutations in PEX1, PEX6, PEX12 and PEX16 that are neither null or missense mutations, including insertions or deletions of single or several nucleotides or whole exon, intronic variants, UTR variants, or biallelic missense mutations in both PEX1 and PEX6.
