# Supplementary material for: Characterization of Severity in Zellweger Spectrum Disorder by Clinical Findings: A Scoping Review, Meta-Analysis and Medical Chart Review
Source: Cells. 2022 Jun 10;11(12):1891. doi: 10.3390/cells11121891 (PMC9221082; doi:10.3390/cells11121891)
Supplement: Supplementary file 1 [file cells-11-01891-s001.zip › cells-1744944-supplementary/Supp_TableS2_ModelResults_NHS.pdf]

	For all ages<br>Probability (95% CI) (%) of being:		
Number of clinical findings*	Severe	Intermediate	Mild
0	0.67 (0.00-1.80)	45.14 (33.82-56.47)	54.19 (42.78-65.60)
1	4.64 (0.00-9.42)	61.19 (50.57-71.82)	34.16 (23.82-44.51)
2	23.56 (11.55-35.56)	60.69 (46.78-74.60)	15.76 (4.76-26.75)
3	63.93 (44.96-82.90)	32.19 (14.60-49.77)	3.89 (0.00-8.84)
4	90.59 (78.92-100.00)	8.91 (0.00-20.04)	0.50 (0.00-1.52)
	For clinical findings presenting <2 years old<br>Probability (95% CI) (%) of being:		
Number of clinical findings*	Severe	Intermediate	Mild
0	0.65 (0.00-1.78)	47.62 (37.44-57.79)	51.74 (41.48-61.99)
1	9.12 (0.36-17.89)	80.64 (66.19-95.08)	10.24 (0.00-23.08)
2	48.12 (26.28-69.96)	51.12 (29.47-72.77)	0.76 (0.00-2.89)
3	88.66 (74.26-100.00)	11.32 (0.00-25.70)	0.02 (0.00-0.11)
4	98.49 (95.17-100.00)	1.51 (0.00-4.83)	0.00 (0.00-0.00)

Supplementary Table S2. Multinomial logistic model results for predicted probabilities of each severity category by number of clinical findings.

*Clinical findings include seizure disorder, abnormal EEG, bilateral renal cortical microcysts and cardiac abnormalities.
