# Supplementary material for: Characterization of Severity in Zellweger Spectrum Disorder by Clinical Findings: A Scoping Review, Meta-Analysis and Medical Chart Review
Source: Cells. 2022 Jun 10;11(12):1891. doi: 10.3390/cells11121891 (PMC9221082; doi:10.3390/cells11121891)
Supplement: Supplementary file 1 [file cells-11-01891-s001.zip › cells-1744944-supplementary/Supp_TableS3_ModelResults_C26-0_NHS.pdf]

	Probability (95% CI) of being:		
**Plasma C26:0 level (g/mL)**	**Severe**	**Intermediate**	**Mild**
0.2	0.21 (0.00-0.64)	7.33 (0.00-15.18)	92.45 (84.40-100.00)
0.26	0.26 (0.00-0.75)	8.54 (0.01-17.08)	91.20 (82.42-99.98)
0.28	0.27 (0.00-0.80)	8.99 (0.22-17.75)	90.74 (81.72-99.76)
0.33	0.32 (0.00-0.92)	10.18 (0.84-19.52)	89.50 (79.88-99.12)
0.34	0.33 (0.00-0.95)	10.44 (0.98-19.89)	89.23 (79.49-98.97)
0.36	0.35 (0.00-1.00)	10.96 (1.28-20.65)	88.68 (78.71-98.66)
0.38	0.37 (0.00-1.06)	11.51 (1.60-21.43)	88.11 (77.90-98.33)
0.39	0.39 (0.00-1.09)	11.80 (1.77-21.82)	87.82 (77.48-98.15)
0.41	0.41 (0.00-1.15)	12.38 (2.13-22.63)	87.21 (76.64-97.77)
0.43	0.44 (0.00-1.21)	12.99 (2.52-23.46)	86.57 (75.77-97.37)
0.45	0.46 (0.00-1.28)	13.63 (2.94-24.32)	85.91 (74.88-96.93)
0.46	0.48 (0.00-1.32)	13.96 (3.16-24.75)	85.56 (74.43-96.70)
0.48	0.51 (0.00-1.39)	14.63 (3.63-25.63)	84.86 (73.51-96.21)
0.51	0.56 (0.00-1.51)	15.69 (4.38-27.00)	83.75 (72.08-95.43)
0.53	0.59 (0.00-1.59)	16.43 (4.93-27.94)	82.98 (71.09-94.86)
0.54	0.61 (0.00-1.63)	16.82 (5.21-28.42)	82.58 (70.59-94.56)
0.55	0.63 (0.00-1.67)	17.20 (5.51-28.90)	82.17 (70.09-94.25)
0.57	0.66 (0.00-1.76)	18.00 (6.12-29.88)	81.34 (69.06-93.61)
0.58	0.68 (0.00-1.81)	18.41 (6.43-30.38)	80.91 (68.54-93.28)
0.6	0.72 (0.00-1.91)	19.25 (7.10-31.40)	80.03 (67.47-92.59)
0.63	0.79 (0.00-2.06)	20.56 (8.15-32.96)	78.66 (65.84-91.47)
0.65	0.83 (0.00-2.17)	21.47 (8.90-34.03)	77.70 (64.71-90.69)
0.66	0.86 (0.00-2.22)	21.93 (9.29-34.57)	77.21 (64.14-90.28)
0.67	0.88 (0.00-2.28)	22.40 (9.68-35.12)	76.72 (63.57-89.86)
0.69	0.93 (0.00-2.39)	23.37 (10.50-36.23)	75.70 (62.40-89.01)
0.77	1.16 (0.00-2.91)	27.49 (14.08-40.90)	71.35 (57.48-85.22)
0.78	1.19 (0.00-2.98)	28.04 (14.56-41.51)	70.78 (56.84-84.71)
0.82	1.32 (0.00-3.27)	30.28 (16.56-44.00)	68.41 (54.22-82.59)
0.86	1.45 (0.00-3.58)	32.61 (18.65-46.57)	65.94 (51.51-80.37)

	Probability (95% CI) of being:		
Plasma C26:0 level (g/mL)	Severe	Intermediate	Mild
0.93	1.72 (0.00-4.17)	36.87 (22.48-51.26)	61.41 (46.55-76.26)
0.94	1.76 (0.00-4.25)	37.50 (23.05-51.95)	60.74 (45.82-75.66)
0.95	1.80 (0.00-4.34)	38.13 (23.61-52.64)	60.07 (45.09-75.05)
0.96	1.85 (0.00-4.44)	38.76 (24.18-53.33)	59.40 (44.35-74.44)
1.02	2.11 (0.00-5.01)	42.61 (27.64-57.57)	55.28 (39.85-70.71)
1.03	2.16 (0.00-5.10)	43.25 (28.22-58.29)	54.59 (39.09-70.09)
1.08	2.40 (0.00-5.61)	46.51 (31.14-61.87)	51.10 (35.27-66.92)
1.16	2.81 (0.00-6.47)	51.69 (35.81-67.58)	45.50 (29.17-61.82)
1.23	3.19 (0.00-7.25)	56.13 (39.86-72.39)	40.68 (24.00-57.36)
1.24	3.25 (0.00-7.37)	56.74 (40.43-73.06)	40.01 (23.29-56.73)
1.29	3.54 (0.00-7.94)	59.78 (43.28-76.27)	36.69 (19.82-53.56)
1.35	3.89 (0.00-8.63)	63.24 (46.64-79.84)	32.87 (15.94-49.79)
1.44	4.44 (0.00-9.68)	68.02 (51.52-84.51)	27.54 (10.84-44.24)
1.49	4.75 (0.00-10.26)	70.42 (54.13-86.71)	24.82 (8.41-41.23)
1.51	4.88 (0.00-10.49)	71.33 (55.15-87.52)	23.79 (7.53-40.05)
1.62	5.57 (0.00-11.72)	75.80 (60.44-91.15)	18.63 (3.48-33.78)
1.63	5.64 (0.00-11.83)	76.16 (60.89-91.42)	18.21 (3.18-33.23)
1.66	5.83 (0.00-12.16)	77.19 (62.22-92.17)	16.98 (2.35-31.62)
1.71	6.15 (0.00-12.71)	78.77 (64.31-93.23)	15.09 (1.15-29.03)
1.75	6.40 (0.00-13.13)	79.90 (65.87-93.94)	13.70 (0.35-27.04)
1.78	6.59 (0.00-13.45)	80.68 (66.97-94.39)	12.73 (0.00-25.61)
1.79	6.65 (0.00-13.56)	80.93 (67.33-94.53)	12.42 (0.00-25.15)
1.94	7.61 (0.13-15.10)	83.89 (71.85-95.94)	8.49 (0.00-18.84)
1.96	7.74 (0.19-15.30)	84.20 (72.34-96.05)	8.06 (0.00-18.09)
2.05	8.32 (0.45-16.19)	85.31 (74.20-96.42)	6.36 (0.00-15.02)
2.08	8.52 (0.55-16.49)	85.60 (74.71-96.50)	5.88 (0.00-14.10)
2.22	9.44 (1.01-17.88)	86.53 (76.36-96.70)	4.03 (0.00-10.39)
2.3	9.98 (1.29-18.67)	86.78 (76.84-96.72)	3.24 (0.00-8.68)
2.31	10.05 (1.33-18.77)	86.80 (76.88-96.72)	3.15 (0.00-8.48)

	Probability (95% CI) of being:		
Plasma C26:0 level (g/mL)	Severe	Intermediate	Mild
2.35	10.32 (1.47-19.18)	86.86 (77.00-96.72)	2.82 (0.00-7.74)
2.42	10.81 (1.73-19.89)	86.87 (77.05-96.68)	2.32 (0.00-6.58)
2.46	11.10 (1.89-20.30)	86.83 (77.01-96.65)	2.08 (0.00-5.99)
2.5	11.38 (2.04-20.72)	86.76 (76.91-96.61)	1.86 (0.00-5.45)
2.59	12.04 (2.40-21.69)	86.51 (76.54-96.48)	1.44 (0.00-4.40)
2.64	12.42 (2.60-22.24)	86.33 (76.25-96.40)	1.25 (0.00-3.90)
2.65	12.49 (2.64-22.35)	86.29 (76.19-96.38)	1.22 (0.00-3.80)
2.78	13.51 (3.16-23.85)	85.65 (75.19-96.11)	0.84 (0.00-2.77)
2.89	14.41 (3.61-25.20)	84.97 (74.12-95.83)	0.62 (0.00-2.11)
3.01	15.44 (4.10-26.77)	84.12 (72.76-95.49)	0.44 (0.00-1.56)
3.2	17.17 (4.85-29.49)	82.58 (70.26-94.90)	0.25 (0.00-0.96)
3.23	17.46 (4.97-29.94)	82.31 (69.82-94.80)	0.23 (0.00-0.89)
3.27	17.84 (5.12-30.57)	81.95 (69.23-94.67)	0.21 (0.00-0.81)
3.37	18.84 (5.49-32.19)	81.01 (67.66-94.35)	0.16 (0.00-0.62)
3.38	18.94 (5.52-32.36)	80.91 (67.50-94.32)	0.15 (0.00-0.61)
3.39	19.04 (5.56-32.52)	80.81 (67.33-94.29)	0.15 (0.00-0.59)
3.54	20.62 (6.07-35.17)	79.29 (64.74-93.83)	0.09 (0.00-0.40)
3.76	23.10 (6.74-39.46)	76.85 (60.49-93.21)	0.05 (0.00-0.22)
4.18	28.37 (7.74-49.01)	71.61 (50.98-92.24)	0.01 (0.00-0.07)
4.59	34.16 (8.54-59.79)	65.83 (40.21-91.45)	0.00 (0.00-0.02)
5.18	43.34 (10.06-76.63)	56.65 (23.37-89.94)	0.00 (0.00-0.00)
5.92	55.46 (14.32-96.59)	44.54 (3.41-85.68)	0.00 (0.00-0.00)
8.8	89.23 (60.13-100.00)	10.77 (0.00-39.87)	0.00 (0.00-0.00)
8.84	89.48 (60.79-100.00)	10.52 (0.00-39.21)	0.00 (0.00-0.00)

Supplementary Table S3. Multinomial logistic model results for predicted probabilities of each severity category by C26:0 fatty acid levels.
